# The microRNA miR-192/215 family is upregulated in mucinous ovarian carcinomas

**DOI:** 10.1038/s41598-018-29332-7

**Published:** 2018-07-23

**Authors:** Antonio Agostini, Marta Brunetti, Ben Davidson, Claes G. Tropé, Ane Gerda Z. Eriksson, Sverre Heim, Ioannis Panagopoulos, Francesca Micci

**Affiliations:** 1 Section for Cancer Cytogenetics, Institute for Cancer Genetics and Informatics, The Norwegian Radium Hospital, Oslo University Hospital, Oslo, Norway; 2 Department of Pathology, The Norwegian Radium Hospital, Oslo University Hospital, Oslo, Norway; 3 Faculty of Medicine, University of Oslo, Oslo, Norway; 4 Department of Gynecology, The Norwegian Radium Hospital, Oslo University Hospital, Oslo, Norway

## Abstract

Different microRNAs are dysregulated in ovarian cancer where some of them have proved to be valid biomarkers. miRNA profiling analyses have shown that the different histotypes of ovarian carcinoma display differential expression of specific miRNAs. In the present study, we used miRNA-sequencing and Real-Time qPCR to detect the expression levels of miRNAs belonging to the miRNA-192/215 family, namely miR-192, miR-194, and miR-215, in different types of ovarian neoplasia, finding that miR-192, miR-194, and miR-215 were upregulated in ovarian carcinomas of the mucinous subtype, but downregulated in other types of carcinoma and in sex cord-stromal tumors. The expression of the said miRNAs was 6-fold higher in mucinous tumors compared to the other histotypes making them candidates for a possible role as diagnostic biomarkers.

## Introduction

MicroRNAs (miRNAs) are non-coding RNAs with diverse biological functions^1^. They play an important regulatory role by targeting specific mRNAs for degradation or translation repression^2^. In so doing, they may influence the development and/or progression of some types of neoplasia as many transcripts are affected simultaneously, leading to profound alteration of signaling pathways^3^.

miRNA deregulation is a pathogenetic mechanism in cancers of the lung^4^, liver^5^, large bowel^6^, and ovaries^7^. Lately, several research groups have aimed to characterize the miRNA signature in the various types of ovarian cancer (OC). The Cancer Genome Atlas consortium launched the first cooperative effort to identify the miRNA profile in high-grade serous ovarian carcinomas^8^ (HGSC), after which many other studies followed focusing on miRNA expression in also the less common OC subtypes^9,10^. Several miRNAs were found to be deregulated^11^ such as miR-141^9^ and miR-192^12^ which are overexpressed exclusively in endometrioid and mucinous carcinomas, respectively. They might hence be diagnostic biomarkers in these tumor subtypes. Also other miRNAs have been shown to be valid prognostic and predictive biomarkers in ovarian carcinomas^11,13^. For example, overexpression of the miR-200 family^14–17^ is correlated with a better response to paclitaxel in patients with OC^18^, and miR-9 upregulation was associated with an improved outcome^19^ and sensitivity to cisplatin^20^. Recently, circulating miRNAs such as miR-21, miR-200c, and miR-1246 were demonstrated in the blood of patients with OC^21,22^.

Despite the increasing interest in the role of miRNA in ovarian carcinogenesis, little is known about this topic in the less common malignant tumors of this site. We therefore investigated the expression status of miRNAs belonging to the miRNA-192/215 family, namely miR-192, miR-194, and miR-215, in different types of ovarian tumors, ranging from sex cord tumors to various carcinomas.

## Results

### Bioinformatic analyses

Downstream analyses of miRNA-seq data gave informative results on 66 samples out of 89. The DESeq. 2 analysis found a mean of 250 miRNAs differentially expressed in each group (data not shown). We narrowed down these results taking into consideration only miRNAs showing more than 4-fold overexpression and Padj minor of 0.005. Using these guidelines/parameters, we found that the miRNAs belonging to the miR-192/215 family were consistently upregulated in mucinous carcinomas (Supplementary Material Table 1). The differential expression analysis for the mucinous group (n = 3 mucinous carcinomas) against all other subgroups (n = 63) gave the following results: miR-192 log2FoldChange of 6.01 (padj = 1.6e^−11^), miR-194 log2FoldChange of 5.9 (padj = 8.86e^−12^), and miR-215 log2FoldChange of 5.8 (padj = 2.6e^−7^).

### Real-Time qPCR analyses

**Figure 1:**
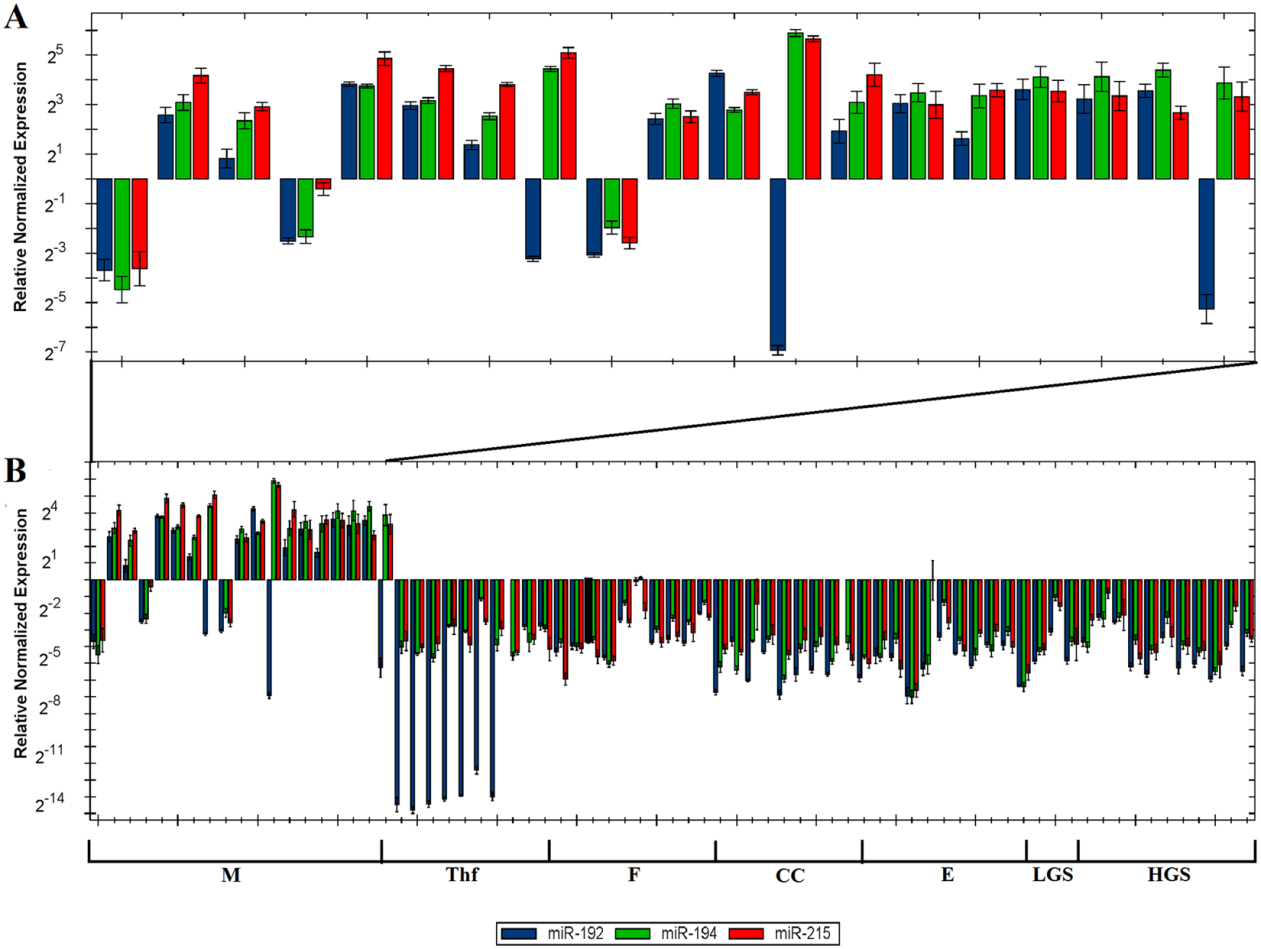
miR-192/215 family expression levels in ovarian tumors assessed by Real-Time qPCR. (**A**) Normalized relative expression of miR-192 (blue), miR-194 (green), and miR-215 (red) in mucinous carcinomas. (**B**) Overview of the normalized relative expression of the miR-192/215 family of miRNAs in the whole series analyzed (73 samples). (M) mucinous carcinoma, (ThF) thecofibroma, (F) fibroma, (CC) clear cell carcinoma, (E) endometrioid carcinoma, (LGS) low-grade serous carcinoma, (HGS) high-grade serous carcinoma.

Since the number of mucinous carcinoma samples analyzed for miRNA differential expression was low (n = 3), we decided to validate the bioinformatic analyses performing Real-Time qPCR for the mentioned miRNAs in an independent cohort of 73 samples from several subgroups of ovarian tumors, i.e., different histological subtypes of carcinoma as well as sex cord-stromal tumors, among which were included samples from 19 mucinous carcinomas. The results obtained by Real-Time qPCR are shown in Fig. 1. At least two of the miRNAs analyzed were upregulated with a mean >6-fold change in most mucinous carcinomas (16 out of 19, 84%; Fig. 1A). The same family of miRNAs was downregulated in all the other types of ovarian tumors (Fig. 1B). Among the mucinous carcinomas, mir-215 showed the highest expression levels (mean = 14.1, median = 11.3) compared to miR-194 (mean = 12.5, median = 8.9) and miR-192 (mean = 5.5, median = 3.8). The thecofibroma group of tumors showed the lowest normalized relative expression level for miR-192 (mean = 0.002, median = 0.00012), whereas fibromas showed considerably higher expression levels (mean = 0.15, median = 0.07). Among ovarian carcinomas, the lowest expression levels for miR-192 were found in the clear cell subgroup (mean = 0.02, median = 0.01) followed by the endometrioid (mean = 0.04, median = 0.04) and low-grade serous carcinoma (LGSC) and HGSC which showed identical values (mean = 0.06, median = 0.03). miR-194 was downregulated in both fibromas (mean = 0.15, median = 0.1) and thecofibromas (mean = 0.11, median = 0.08). MiR-194 was highly downregulated in clear cell (mean = 0.04, median = 0.04) and endometrioid carcinomas (mean = 0.08, median = 0.04), whereas the two serous carcinoma subgroups showed moderate downregulation (LGSC: mean = 0.2, median = 0.1; HGSC: mean = 0.11, median = 0.9). miR-215 was downregulated in both sex-cord tumors and carcinomas (see Table 1 for expression levels). Real-time qPCR for the thymidylate synthetase (*TYMS*), Zinc finger E-box-binding homeobox 2 (*ZEB2*), and Mouse double minute 2 homolog (*MDM2*) genes was performed as these genes are known to be targeted and regulated by the miR-192/215 family^23^. The expression levels of the three mentioned genes were similar in all ovarian tumors included in our series. No significant inverse correlation was found between the expression of these genes and expression of miR-192, miR-194, and miR-215 in the mucinous carcinomas or in the other groups of ovarian tumors analyzed (data not shown).

**Table 1:** miR-215 normalized relative expression in ovarian tumors.

Hystotype	Mean	Median
Fibroma	0.09	0.07
Thecofibroma	0.09	0.07
Mucinous	14.1^a^	11.3
Endometrioid	0.1	0.07
Clear cell	0.1	0.07
Low-grade Serous	0.15	0.08
High-grade Serous	0.09	0.07

^a^Normalized relative expression assessed with the 2^−ΔΔCt^ (Livak) method using two commercially available normal ovarian controls as reference.

## Discussion

Several studies have identified different miRNA signatures in the various types of OC and some of these miRNAs are successfully used as diagnostic and prognostic markers^17,24,25^. miR-200a and miR-200c were found upregulated in serous, endometrioid, and clear cell carcinomas, whereas miR-200b and miR-141 overexpression was characteristic of the endometrioid and serous histotypes^9,26^. Moreover, endometrioid carcinomas have shown upregulation of three additional miRNAs, namely miR-21, miR-203, and miR-205^9^. Calura *et al*.^12^ found the clear cell histotype to be characterized by a higher expression of miR-30a whereas mucinous tumors had higher levels of miR-192 and miR-194^12^. Our results are in accordance with previous findings inasmuch as both miR-192 and miR-194 were found overexpressed in mucinous carcinomas but downregulated in the other histotypes. However, we found also another miRNA belonging to the same family upregulated in the majority of mucinous carcinomas, miR-215. This further emphasizes the specificity of the expression profile of this miRNA family in mucinous ovarian carcinomas and highlights the possibility that these molecules may be used as diagnostic biomarkers.

We report for the first time the extent to which miR192/215 is expressed in sex-cord stromal tumors showing that all three miRNAs are downregulated similarly to what is seen in all OC histotypes with mucinous carcinomas as the sole exception. The miR192/215 family is known to be downregulated in different malignancies such as renal cell carcinoma^23^, colorectal cancer^27^, and multiple myeloma^28^. Our analysis showed variable expression of miRNAs in the mucinous tumors; more precisely, in three carcinomas, all three miRNAs were down-regulated, two tumors showed overexpression of miR-194 and miR-215 but downregulation of miR-192, and the last three carcinomas showed no expression of the three miRNAs. We checked if the observed expression differences among the mucinous samples correlated with parameters such as tumor stage, grade, recurrence, or time of death, but found nothing (Table 2). However, the three cases showing miRNA downregulation did present some special features such as a mixed mucinous and endometrioid histotype in case 1, atypia in case 4, and neuroendocrine differentiation in case 9 (Table 2). Our results further revealed that, besides general downregulation of miR-192 in sex cord-stromal tumors, fibromas and thecofibromas differed in their expression levels: expression of miR-192 was ten times higher in fibromas than in thecofibromas.

**Table 2:** Mucinous carcinoma samples overview.

Sample	Status	Metastasis	Treatment	Stage	Grade	Diagnosis	Specific immunostaining
1	alive without disease	none	carboplatin/paclitaxel	IC	BOT	Endometrioid adenocarcinoma with focal mucinous differentiation	mCEA+/− (extracellular), ER+, PAX8+
2	dead, unknown reason	NA	carboplatin/paclitaxel	NA	1	Mucinous adenocarcinoma	CK7+, CK20+/−, PAX8+
3	alive without disease	none	none	IA	BOT	Mucinous cystadenoma	mCEA+, CK7+, CK20+, PAX8+
4	dead of disease	recurrence	oxaliplatin and Xeloda, 5FU	IIIB	1	Mucinous adenocarcinoma	CK7+, CK20−, PAX8+
5	dead of disease	pelvic tumor	carboplatin/paclitaxel	IIIC	NA	Mucinous adenocarcinoma of uncertain origin in the ovary	mCEA+, CK7 focally+, CK20 focally+, PAX8−
6	dead of disease	pelvic tumor	carboplatin/paclitaxel	IIIC	NA	Mucinous adenocarcinoma of uncertain origin in the ovary	PAX8−
7	dead of disease	recurrence	palliative	NA	NA	Low-grade mucinous carcinoma	Archival slides unavailable for reassessment
8	dead of disease	pelvic tumor, liver	none	IA	BOT	Mucinous carcinomas	CK7+, CK20−, PAX8+
9	dead of other reason	none	none	IC	2	Mucinous adenocarcinoma with neuroendocrine differentiation	mCEA+/−, CK7+, CK20−, PAX8+, Synaptophysin+/−, Chromogranin A+/−
10	dead of disease, after recurrence	residual tumor	none	IIIC	2	Mucinous adenocarcinoma of uncertain origin in the ovary	mCEA+, CK7+, CK20+, CDX2+/−, PAX8−
11	alive without disease	none	none	IA	1	Mucinous adenocarcinoma	mCEA+, CK7+, CK20+/−, PAX8−, CDX2−, ER−, PR−
12	alive without desease	none	none	IIC	1	Mucinous adenocarcinoma	mCEA+/−, CK7+, CK20+/−, PAX8+, CDX2+/−, CA 125−
13	Alive without disease	rectum	Adjuvant Oxaliplatin and Xeloda	IIB	NA	Mucinous adenocarcinoma	CEA+, CK7+, CK20+, CDX2+/−
14	Alive without disease	none	none	IA	1	Mucinous adenocarcinoma	CEA+, CK7+, CK20−, CDX2−
15	Alive without disease	none	none	IA	NA	Mucinous adenocarcinoma	Archival slides unavailable for reassessment
16	Alive with disease	pelvic tumor	HIPEC with MitomycinC	IVb	2 with neuroendocrine differentiation	Mucinous adenocarcinoma	CEA+, CK7+, CK20+, CDX2+, Vilin
17	Dead of disease	pelvic tumor	none	IA	1	Mucinous adenocarcinoma	Archival slides unavailable for reassessment
18	Alive without disease	pelvic tumor	none	IA	1	Mucinous adenocarcinoma	Archival slides unavailable for reassessment
19	Alive without disease	pelvic tumor	none	IC	1	Mucinous adenocarcinoma	CK7+, CK20+, CDX2+, PAX8+

It is presently unknown what lies behind the different expression profiles within the miR192/215 family of miRNAs, if it is attributable to a genomic rearrangement such as loss of chromosomal material from 1q41 and 11q13 (where these miRNAs are located) or if some epigenetic silencing mechanism is operative. Additional studies should shed more light on the importance of these pathways.

Previous studies showed the miR-192/215 family of miRNAs to be downregulated in renal cell carcinoma^23^ and multiple myeloma^28^. When expression of this family of miRNAs is restored in these malignancies, the miRNAs act as tumor supressors repressing the oncogenes *TYMS*, *ZEB2*, and *MDM2*^23,28,29^. In order to gain new insights into the pathogenesis of mucinous carcinomas in particular, and ovarian carcinomas in general, we checked the expression status of the aforementioned three genes targeted by the miR-192/215 family, expecting to find different expression patterns in mucinous carcinomas compared to the other groups of ovarian tumors analyzed. Interestingly, the mean expression of the three genes (*TYMS*, *ZEB2*, and *MDM2*) was upregulated in all ovarian tumor subtype analyzed. These results suggest that also other mechanisms than miRNAs are active in the regulation of these genes in ovarian tumors. Recently, Zhang *et al*.^30^ showed that miR-192/215 can also act as oncomirs promoting epithelial-mesenchymal transition in gastric cancer repressing the tumor suppressor gene nonsense mediated mRNA decay associated PI3K related kinase (*SMG1*). These findings suggest that the miR192/215 family miRNAs may exert oncogenic functions in mucinous carcinomas.

## Material and Methods

### Tumor material

**Figure 2:**
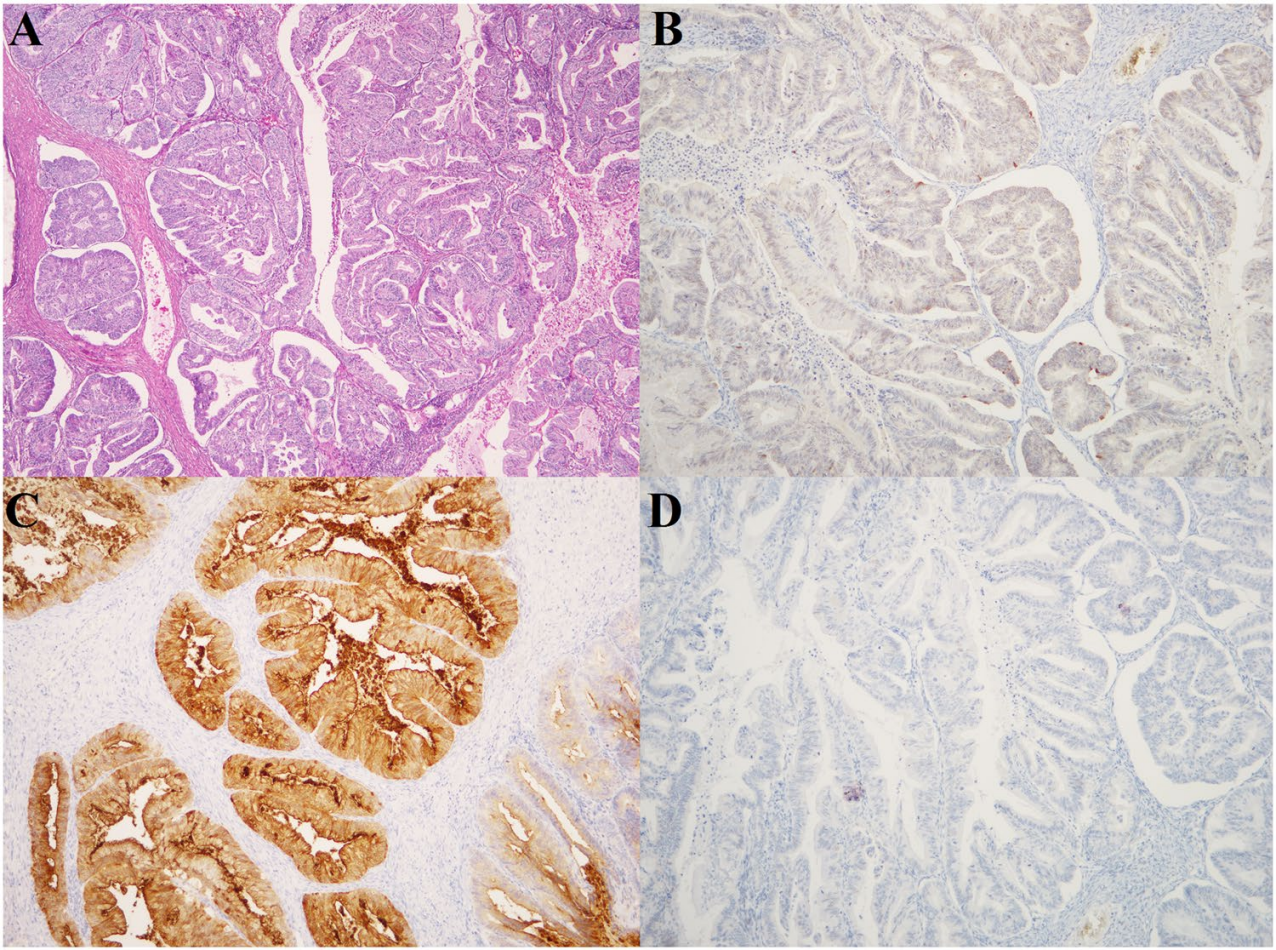
Immunohistochemistry analyses of case XI. (**A**) Staining for Hematoxylin and Eosin (HE) and immunostaining for PAX8 (negative) (**B**), CEA (positive) (**C**), and ER (negative) (**D**).

The material consisted of 155 fresh frozen samples from ovarian tumors surgically removed at The Norwegian Radium Hospital from 1998 to 2008. Eighty-nine samples (53 high-grade serous carcinomas (HGSC), 17 endometrioid carcinomas, 7 low-grade serous carcinomas (LGSC), 5 clear cell carcinomas, 4 borderline tumors, and 3 mucinous carcinomas) were investigated by means of next generation sequencing (NGS; miRNA sequencing, see below). Sixty-six other samples (12 mucinous carcinomas, 10 clear cell, 10 endometrioid, 10 HGSC, and 4 LGSC as well as 10 thecofibromas and 10 fibromas) were used as an independent series to validate the results from NGS and bioinformatic analyses. As part of this validation, 12 mucinous carcinomas were yet again reexamined by an expert pathologist, immunostained for CK7 and CK20, and additional staining was performed for PAX8 to identify the primary versus metastatic origin of the ovarian tumor (Table 2, Fig. 2). Seven additional mucinous carcinomas were retrieved from the Pathology biobank to further validate the data regarding this subtype (Table 2). The study was approved by the regional ethics committee (Regional komité for medisinsk forskningsetikk Sør-Øst, Norge, http://helseforskning.etikkom.no), project numbers 2.2007.425 and 2011/2071. All methods were performed in accordance with the guidelines and regulations approved by the institutional review board (protokollutvalget - Radiumhospitalet). Written informed consent was obtained from the patients.

### Total RNA extraction

Total RNA was extracted using miRNeasy Kit (Qiagen, Hilden, Germany) and QIAcube (Qiagen) according to the manufacturers’ recommendations. RNA concentration and purity was measured using a Nanovue Spectrophotometer (GE Healthcare, Pittsburgh, PA, USA).

### miRNA sequencing

miRNA sequencing was performed on 89 samples at the Norwegian Sequencing Center at Ullevål Hospital Oslo (https://www.sequencing.uio.no/). The sequencing library was created using the ScriptMiner Small RNA-seq Library Preparation Kit (Epicentre, Madison, WI, USA). RNA was sequenced using an Illumina HiSeq. 2500 instrument and the Illumina software pipeline was used to process image data into raw sequencing data. Only sequence reads marked as “passed filtering” were used in the downstream data analysis.

### Bioinformatic Analyses

The raw data were trimmed using Cutadapt^31^ and the quality of the reads was checked with FastQC (http://www.bioinformatics.babraham.ac.uk/projects/fastqc/). The reads were aligned using the alignment program STAR^32^. The miRNA detection rate was assessed for each sample by correlating the number of reads mapped to reference miRNAs obtained from a freely available miRNA database (http://www.mirbase.org/) and the number of detected miRNAs. An arbitrary cutoff was set at 1.5 million mapped reads as suggested by Metpally *et al*.^33^, and all samples whose reads were below that threshold were removed from further analyses (13 out of 89). Finally, the read counts were analyzed for differential expression using the Bioconductor package DESeq. 2^34^ comparing each tumor histotype against all the others. Only results with a padj minor of 0.005 were considered.

### Real-Time Polymerase Chain Reaction (Real-Time PCR)

The expression of the miRNAs and genes analyzed in this study was assessed with Real-Time qPCR on 73 samples. The PCR analyses were performed using the CFX96 Touch Real-Time PCR detection system (Bio-Rad Laboratories, Oslo, Norway). The reactions were carried out in quadruplicate using the TaqMan Universal Master Mix II with no UNG (Applied Biosystems, Foster City, CA, USA) following the manufacturer’s protocol. Human Universal Reference Total RNA (Clontech, Mountain View, CA, USA) was used as internal reaction control, whereas two commercially available controls, MVP Total RNA Human Ovary (Agilent Technologies, Santa Clara, CA, USA) and Human Ovary Total RNA (Zyagen, San Diego, CA, USA), were used as reference for relative expression normalization. The Real-Time data were analyzed with Bio-Rad CFX manager 3.1 (Bio-Rad). The normalized expression was calculated using the 2^−ΔΔCt^ (Livak) method^35^.

### miRNA expression

Ten ng of total RNA were reverse transcribed with the TaqMan MicroRNA Reverse Transcription Kit (Applied Biosystems) following the manufacturer’s protocol. miRNA expression was assessed with Real-Time qPCR using the TaqMan MicroRNA assays (Applied Biosystems) for miR-192 (000491), miR-194 (000493), and miR-215 (000518). The *RNU6B* gene (TM:001093) was used as reference as it is stably expressed in ovarian tumors (all subgroups)^36^. The mean Cq values of *RNU6B* for each sample were checked to be in the range 26–27 before performing the relative normalized expression to avoid bias in the expression analysis.

### Gene expression

One µg of extracted total RNA for each tumor was reverse-transcribed in a 20 μl reaction volume using iScript Advanced cDNA Synthesis Kit according to the manifacturer’s instructions (Bio-Rad Laboratories, Oslo, Norway). Gene expression was assessed with Real-Time PCR using the TaqMan Gene Expression Assays (Applied Biosystems) for *ZEB2* (Hs00207691_m1), *MDM2* (Hs00540450_s1), and *TYMS* (Hs00426586_m1). *RPL4* (Hs_01939407_gH) was used as a reference gene because it shows stable expression in ovarian cells^37^.

## Electronic supplementary material

Supplementary Dataset 1
