# Supplementary material for: The microRNA miR-192/215 family is upregulated in mucinous ovarian carcinomas
Source: Sci Rep. 2018 Jul 23;8:11069. doi: 10.1038/s41598-018-29332-7 (PMC6056508; doi:10.1038/s41598-018-29332-7)
Supplement: Supplementary file 1 — Supplementary Dataset 1 [file 41598_2018_29332_MOESM1_ESM.docx]

**The microRNA miR-192/215 family is upregulated in mucinous ovarian carcinomas**

**Antonio Agostini^1^, Marta Brunetti^1^, Ben Davidson^2,3^, Claes G. Tropé^4^, Sverre Heim^1,3^, Ioannis Panagopoulos^1^, Francesca Micci^1^**

1 Section for Cancer Cytogenetics, Institute for Cancer Genetics and Informatics, The Norwegian Radium Hospital, Oslo University Hospital, Oslo, Norway

2 Department of Pathology, The Norwegian Radium Hospital, Oslo University Hospital, Oslo, Norway

3 Faculty of Medicine, University of Oslo, Oslo, Norway

4 Department of Gynecology, The Norwegian Radium Hospital, Oslo University Hospital, Oslo, Norway

**Supplementary Material Table 1. Data of differential expression analyses in mucinous carcinoma.**

| **miRNA** | **baseMean** | **log2FoldChange** | **lfcSE** | **stat** | **pvalue** | **padj** |
| --- | --- | --- | --- | --- | --- | --- |
| hsa-miR-194-5p | 4828.33578 | 5.969483318 | 0.772272 | 7.72977 | 1.08E-14 | 8.86E-12 |
| hsa-miR-192-5p | 8511.79199 | 6.019279838 | 0.791328 | 7.60655 | 2.82E-14 | 1.16E-11 |
| hsa-miR-215-5p | 83.9518552 | 5.886917243 | 0.962762 | 6.114613 | 9.68E-10 | 2.65E-07 |
| hsa-miR-708-5p | 3632.12253 | -4.650864338 | 0.85789 | -5.42128 | 5.92E-08 | 1.22E-05 |
| hsa-miR-708-3p | 4080.95499 | -4.640876659 | 0.907331 | -5.11487 | 3.14E-07 | 5.16E-05 |
| hsa-miR-192-3p | 24.6197413 | 5.093357762 | 1.039546 | 4.899598 | 9.60E-07 | 0.000132 |
| hsa-miR-100-5p | 22433.6067 | -3.835902712 | 0.803233 | -4.77558 | 1.79E-06 | 0.000191 |
| hsa-miR-3120-3p | 186.980819 | -6.966748392 | 1.461032 | -4.76837 | 1.86E-06 | 0.000191 |
| hsa-miR-125b-5p | 80487.6474 | -3.584653147 | 0.845923 | -4.23756 | 2.26E-05 | 0.001857 |
| hsa-miR-125b-1-3p | 172.489653 | -3.617706403 | 0.870894 | -4.15401 | 3.27E-05 | 0.002441 |
| hsa-miR-204-5p | 331.949459 | -4.286746881 | 1.081523 | -3.96362 | 7.38E-05 | 0.004668 |
| hsa-miR-199b-5p | 3244.50288 | -2.88620102 | 0.758389 | -3.8057 | 0.000141 | 0.008302 |
| hsa-miR-199a-3p | 7596.86494 | -2.851679864 | 0.768441 | -3.711 | 0.000206 | 0.009982 |
| hsa-miR-199b-3p | 7596.86494 | -2.851679864 | 0.768441 | -3.711 | 0.000206 | 0.009982 |
| hsa-miR-503-5p | 503.439746 | 2.846019208 | 0.795194 | 3.579023 | 0.000345 | 0.015005 |
| hsa-miR-887-3p | 257.061282 | -2.416927658 | 0.675581 | -3.57756 | 0.000347 | 0.015005 |
| hsa-miR-10b-5p | 129899.254 | -3.070129651 | 0.867852 | -3.53762 | 0.000404 | 0.016594 |
| hsa-let-7c-5p | 6569.59575 | -2.892392886 | 0.824871 | -3.50648 | 0.000454 | 0.016966 |
| hsa-miR-934 | 225.401552 | -4.914831096 | 1.398823 | -3.51355 | 0.000442 | 0.016966 |
| hsa-miR-10b-3p | 417.781608 | -2.781901738 | 0.814107 | -3.41712 | 0.000633 | 0.020809 |
| hsa-miR-146b-5p | 43126.4763 | -2.643828088 | 0.793414 | -3.33222 | 0.000862 | 0.027239 |
| hsa-miR-194-3p | 7.01964437 | 4.522279798 | 1.366126 | 3.310296 | 0.000932 | 0.02736 |
| hsa-miR-125b-2-3p | 657.988702 | -2.619179567 | 0.802708 | -3.26293 | 0.001103 | 0.030213 |
| hsa-miR-885-5p | 81.5337569 | -4.630401356 | 1.418135 | -3.26514 | 0.001094 | 0.030213 |
| hsa-miR-146b-3p | 106.62141 | -2.623476502 | 0.810147 | -3.23827 | 0.001203 | 0.031887 |
| hsa-miR-130a-3p | 15627.1035 | -1.923411998 | 0.602496 | -3.19241 | 0.001411 | 0.036243 |
| hsa-miR-100-3p | 22.6506647 | -3.583651889 | 1.136248 | -3.15394 | 0.001611 | 0.038945 |
| hsa-miR-99a-5p | 186857.734 | -2.82954877 | 0.896629 | -3.15576 | 0.001601 | 0.038945 |
| hsa-miR-218-5p | 6524.59153 | -2.487885356 | 0.798244 | -3.1167 | 0.001829 | 0.042887 |
| hsa-miR-615-3p | 132.314106 | -2.865527597 | 0.921736 | -3.10884 | 0.001878 | 0.042887 |
| hsa-miR-3120-5p | 111.753852 | -3.102925965 | 1.015938 | -3.05425 | 0.002256 | 0.048405 |
| hsa-miR-7641 | 112.563923 | 2.77717599 | 0.91315 | 3.041315 | 0.002355 | 0.048405 |
| hsa-miR-7704 | 1111.55161 | 1.750670956 | 0.574988 | 3.04471 | 0.002329 | 0.048405 |
| hsa-miR-99a-3p | 300.44937 | -2.4536717 | 0.805922 | -3.04455 | 0.00233 | 0.048405 |
| hsa-miR-199a-5p | 14989.5189 | -2.47340077 | 0.816277 | -3.0301 | 0.002445 | 0.049014 |
